# Effect of Resveratrol and Curcumin on Gene Expression of Methicillin-Resistant *Staphylococcus aureus* (MRSA) Toxins

**DOI:** 10.4014/jmb.2309.09001

**Published:** 2023-11-15

**Authors:** Areej M. El-Mahdy, Maisa Alqahtani, May Almukainzi, Majed F. Alghoribi, Shaymaa H Abdel-Rhman

**Affiliations:** 1 Department of Microbiology and Immunology, Faculty of Pharmacy, Mansoura University, Mansoura 35516, Egypt; 2 Biology Department, College of Science, Princess Nourah bint Abdulrahman University, P.O. Box 84428, Riyadh 11671, Saudi Arabia; 3 Department of Pharmaceutical Sciences, College of Pharmacy, Princess Nourah bint Abdulrahman University, P.O. Box 84428, Riyadh 11671, Saudi Arabia; 4 Infectious Diseases Research Department, King Abdullah International Medical Research Center, Riyadh, Saudi Arabia; 5 King Saud bin Abdulaziz University for Health Sciences, Riyadh, Saudi Arabia; 6 Department of Pathology and Laboratory Medicine, King Abdulaziz Medical City (KAMC), Ministry of National Guard Health Affairs (MNGHA), Riyadh, Saudi Arabia; 7 Department of Pharmaceutics and Pharmaceutical Biotechnology, Faculty of Pharmacy, Taibah University, AlMadinah Al Munawwarah, Saudi Arabia

**Keywords:** *Staphylococcus aureus*, Methicillin-Resistant *Staphylococcus aureus* (MRSA), resveratrol, curcumin, toxins, gene expression

## Abstract

*Staphylococcus aureus* is an opportunistic pathogen that can lead to a number of potentially terrible community- and hospital-acquired illnesses. Among the diverse set of virulence factors that *S. aureus* possesses, secreted toxins play a particularly preeminent role in defining its virulence. In this work, we aimed to facilitate the development of novel strategies utilizing natural compounds to lower *S. aureus*’s toxin production and consequently enhance therapeutic approaches. Two natural polyphenols, resveratrol (RSV) and curcumin (CUR), were tested for their effect on reducing toxin gene production of MRSA isolates. Fifty clinical MRSA isolates were gathered from Riyadh and Jeddah. Molecular screening of toxin genes (*sea*, *seb*, *sec*, *sed*, *seh*, *lukF*, and *lukS*) harbored by MRSA was performed. Sub-inhibitory concentrations of RSV (50 μg/ml) and CUR (20 μg/ml) were determined to study their effect on the gene expression MRSA’s toxin genes. Our findings revealed the presence of the tested genes in MRSA isolates, with *lukF* being the most prevalent gene and *seh* the least detected gene. We found that RSV reduced the relative expression of toxin genes, *sea*, *seb*, *lukF*, and *lukS*, respectively, while CUR decreased the relative expression of *sea* and *seb* genes in the examined isolates. Regarding *lukF* and *lukS*, CUR downregulated the expression of both genes in some isolates and upregulated the expression in other isolates. From these results, we concluded that RSV and CUR could be used as alternative therapeutic approaches to treat MRSA infections through reducing toxin production.

## Introduction

*Staphylococcus aureus* is a gram-positive opportunistic bacterium that is often found as normal flora. Sometimes it enters the body through the skin and causes both community- and hospital-acquired infections. These infections are of three categories; infections of the skin, disease through toxin production, or systemic and life-threatening conditions. Toxin-mediated and invasive infections depend mainly on strains and sites of infection [1, 2].

In particular, the widespread antibiotic resistance among *S. aureus* isolates makes their infection problematic. In comparison to methicillin-sensitive *S. aureus* (MSSA), methicillin-resistant *S. aureus* (MRSA) has been linked to higher rates of hospitalization and death. MRSA strains have developed resistance to other antibiotics, which limits the available treatment options [3, 4].

*S. aureus* has established a regulated toxin production system in which the three main classes generated are pore-forming toxins, exfoliative toxins, and superantigens. There is a distinct relationship between these toxins and some life-threatening conditions. Panton-Valentine leucocidin (PVL) is a powerful staphylococcal exotoxin that exerts its activity through two secretory proteins; types F and S. In addition to causing polymorphonuclear cells to release oxygen metabolites and lose their plasma membrane, PVL also causes lysozyme production and the release of histamine from human basophils. PVL determinants are found in the majority of *S. aureus* strains that cause necrotizing pneumonia and primary cutaneous infections. These toxins are found in 85% of the community-acquired MRSA strains [5]. On the other hand, PVL genes were not detected in any *S. aureus* strains causing endocarditis, hospital-acquired infections, toxic-shock syndrome, or urinary tract infections [6].

The second class, serine proteases known as exfoliative toxins (ETs), bind to and hydrolyze desmosome cadherins in the epidermis' outermost layers [7]. They cleave keratinocyte junctions in the epidermis of the skin leading to peeling and blistering of the skin. The principal ETs are ETA, ETB, ETC, and ETD, and these are secreted by only 5% of strains. The most common ones that cause skin injury in humans are ETA and ETB, while ETC has only been linked to infections in horses, and ETD was first discovered in a clinical sample of *S. aureus* in 2002 [8].

Superantigens (SAgs), formerly known as staphylococcal enterotoxins (SEs), are the third class and are the cause of food poisoning caused by *S. aureus* [5]. In addition to the toxic shock syndrome toxin (TSST-1) and the staphylococcal enterotoxins, there are currently 11 staphylococcal superantigen-like (SSL) toxins and 23 staphylococcal SAg toxins [9].

Numerous secondary metabolites produced by plants act as a chemical barrier against infections; therefore, it makes sense to use them as a source of prototype chemicals. Natural phenolic substances have antimicrobial properties in part because they harm bacterial membranes, prevent the production of virulence factors including enzymes and toxins, and hinder the growth of biofilms [10].

Red wine, grapes, berries, peanuts, and other foods naturally contain the phytoalexin RSV (3, 5, 4'-trihydroxy-trans-stilbene) that is also produced due to pathogen attack [11]. RSV, a polyphenolic substance, has potent anti-estrogenic, antioxidant, antiarteriosclerosis, antibacterial, and anticancer characteristics, as well as the capacity to prevent the development of hepatic fat and eicosanoids [12, 13].

Curcumin (CUR) is a natural chemical obtained from a variety of *Curcuma* species as it is one of the major components of turmeric roots/rhizomes (*Curcuma longa* L.) [12]. Studies have shown that curcumin, the main polyphenolic curcuminoid in turmeric, contains antioxidant, anticarcinogenic, anti-HIV, antibacterial, and anti-inflammatory properties [14].

According to several studies from the past ten years, combining naturally occurring compounds produced from plants with widely used antibiotics may represent a new approach that might be utilized to defeat bacteria that are resistant to several antibiotics. Promising natural antimicrobial agents are plant-derived polyphenols, which have been demonstrated to exhibit antibacterial activity and to make antibiotics more effective against multidrug-resistant strains [15]. Few studies have shown the effect of RSV on the gene expression of MRSA enterotoxins and PV-leukocidins. Moreover, the effect of CUR on gene expression in MRSA was not discussed in any of the previous studies. In this research, we sought to examine the impact of RSV and CUR, two naturally occurring polyphenolic compounds, on the toxin production by MRSA isolates.

## Materials and Methods

### Bacterial Strains and Reagents

A total of 50 MRSA isolates were acquired from KAIMARC, Riyadh, and Jeddah, Kingdom of Saudi Arabia (KSA). They were obtained from various clinical samples of the following origin: respiratory, wound, tissue, eye swab, bone sterile, aspiration fluid, bed swab, ear, nasal swab, and groin. The VITEK 2 system (bioMerieux, France) and *S. aureus* (Baa977) as a reference strain were used to identify and confirm that each isolate was MRSA. Confirmed isolates were kept at −80°C for further studies.

RSV (cat. no. 501-36-0, Hi-Tech Development Zone, Xi’an, China) and CUR (cat. no. 458-37-7, AK Scientific, USA) were used for the preparation of stock solutions (12 mg/ml) in dimethyl sulfoxide (DMSO) (Merck, Sigma Aldrich, USA). The working solutions were prepared just before each experiment. Negative-control cultures received an equivalent amount of DMSO. This research was approved by the Institutional Review Board, Princess Nourah bint Abdulrahman University.

### Determination of Minimum Inhibitory Concentration (MIC) of RSV and CUR

The procedures of the Clinical Laboratory Standards Institute (CLSI) were followed with minor modifications to estimate the MIC of RSV and CUR against MRSA isolates [16]. RSV and CUR were diluted two-fold serially (2000 to 15.6 g/ml) and added to overnight cultures of strains in Brain Heart Infusion (BHI) broth (HiMedia, India) that were adjusted to an optical density at 600 nm of 0.08 to 0.1 in a 96-well microtiter plate. The lowest concentration at which the organism shows no discernible growth was determined to be the MIC after incubation at 37°C. All experiments were done in triplicate.

### Effect of Sub-MIC on the Viability of MRSA

To ascertain the effects of RSV (100 and 50 μg/ml) and CUR (50 and 20 μg/ml) sub-MICs on MRSA growth, viable count experiment using pour plate method was performed [17]. The number of surviving cells of MRSA isolates treated with sub-MICs of RSV and CUR was compared with the untreated cells cultivated under the same conditions.

### Molecular Characterization of Toxin-Encoding Genes

All MRSA strains had their genomic DNA extracted using a DNA Mini Extraction Kit (Qiagen, Germany) according to the manufacturer's instructions.

The existence of several toxin genes (*sea*, *seb*, *sec*, *sed*, *seh*, *lukF*, and *lukS*) [18] [19] was investigated using uniplex PCR amplifications. Table 1 lists sets of primers used in this study. A thermocycler machine (Cleaver Scientific Ltd., GTC96S, UK) was used to carry out the amplifications. For each primer, the PCR was run under predetermined temperature profile and PCR conditions [18, 19]. The PCR products were examined on 1.2%agarose gel (GIBCO Bethesda Research Lab, Life Technology, USA) stained with ethidium bromide (Sigma No. E7637), compared with 100 bp plus DNA ladder (Thermo Fisher Scientific, USA) and scanned using a gel documentation and analysis system (Gel DocTM XR^+^, Bio-Rad, USA). Strains that produced the PCR products were used for further experiments.

### Quantitative Real-Time PCR

Stored bacterial isolates were subcultured on BHI agar at 37°C for 18–24 h. For each isolate, 3 bacterial suspensions were prepared and adjusted to 0.5 MacFarland in 3 tubes; one of them was used as the control (C) without the phenolic compound and the other two tubes (T) were treated with the chosen sub-MIC of RSV (50 μg/ml) and CUR (20 μg/ml). The (C) and (T) tubes were incubated overnight on a shaker incubator at 37°C and 150 rpm and centrifuged before mechanical disruption. TRI Reagent (T9424 Sigma-Aldrich, USA) was used to extract the total RNA. RNA concentration and purity were calculated using a Nanodrop-1000 Spectrophotometer (USA). The RNA integrity was confirmed by gel electrophoresis using 2% agarose gel loaded with 5 μl of the total RNA.

The High-Capacity cDNA Reverse Transcription Kit (cat. no. 4368814, Thermo Fisher) was used to reverse-transcribe whole RNA according to the manufacturer's instructions. The cDNA was stored at -20°C.

Using the previously described primers, RT-PCR was used to determine the expression level of the *sea*, *seb*, *LukF*, and *LukS* genes. *nuc* gene served as an internal MRSA housekeeping gene [20]. To prepare the reaction mixture, HOT FIREPol SolisGreen qPCR Mix (5X, cat. no. 08-46-0000S) was used; and the program was run as follows: PCR initial activation step for 10 min at 95°C, denaturation for 30 s at 95°C, annealing for 30 s at suitable annealing temperature for each primer, and extension at 72°C for 30 s. Reactions were run for 45 cycles using Quantstudio 5 (Applied Biosystems, Thermo Fischer).

Melting curve analysis of the PCR product(s) to verify their specificity and identity was performed and the 2^−ΔΔCT^ method was used to calculate the normalized relative expression.

### Statistical Analysis

GraphPad Prism 5 by Dotmatics was used to analyze the results. The treated isolates were compared with the control group using one-way analysis of variance (ANOVA) and Dunnett's posttest for multiple comparisons. Statistical significance was defined as a *p*-value of 0.05 or less.

## Results

### Determination of MIC and the Effect of Sub-MICs of Resveratrol and Curcumin on the Viability of MRSA Strains

To calculate the impact of their sub-MICs on bacterial growth and toxin expression, the microbroth dilution method was used to determine the MICs of both RSV and CUR. The MICs were found to be 500 and 125 μg/ml, respectively, for all the tested isolates for RSV and CUR. When cultured with 50 μg/ml RSV and 20 μg/ml CUR, all isolates exhibited nearly the same bacterial count (158 × 10^8^ CFU/ml) compared to that of the untreated cultures (162 × 10^8^ CFU/ml). Therefore, in further experiments, concentrations of 50 and 20 μg/ml of RSV and CUR, respectively, were used to assess the virulence factors of MRSA isolates.

### Molecular Screening of Various Toxin Genes Among MRSA Strains

**Fig. 1:**
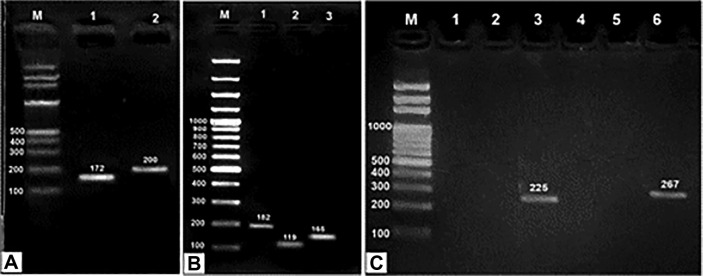
PCR for toxin genes in MRSA isolates. (**A**) Isolate No. 2: Lane (1) *sea*, Lane (2): *seb*. (**B**) Lane (1) *sec* (Isolate No. 7), Lane (2): *sed* (Isolate No. 17), Lane (3): *she* (Isolate No. 10). (**C**) Isolate No. 4: Lane (3) *lukF*, Lane (6): *lukS*. Lane (M): 100 bp plus DNA ladder.

**Fig. 2:**
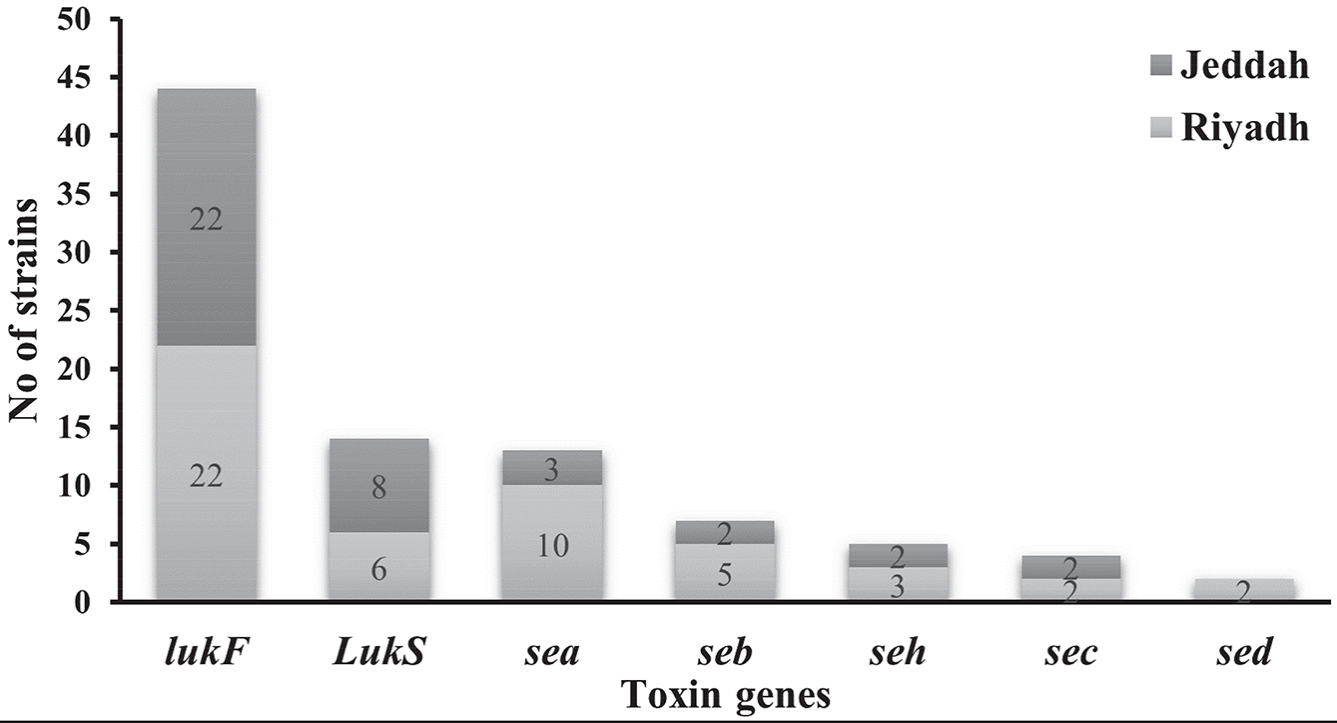
Distribution of toxin genes among MRSA strains acquired from KAIMARC, Riyadh and Jeddah, KSA.

Fig. 1 represented the molecular detection of toxin genes (*sea*, *seb*, *sec*, *sed*, *seh*, *lukF*, and *lukS*) that was performed by uniplex PCR. *lukF* gene was found in 44 strains (88%) with equal distribution between strains from Riyadh and Jeddah (22 isolates from both harbored *lukF* gene). The distribution of other toxin genes was as follows: *lukS* gene was detected in 14 strains (28%), followed by *sea* gene in 13 strains (26%), *seb* in 7 strains (14%), *seh* in 5 strains (10%), *sec* in 4 strains (8%), and *sed* in only 2 strains (4%), as shown in Fig. 2. Table 2 displays the 11 toxin gene patterns that were identified through examination of the PCR results. Five toxicity patterns were exhibited by strains isolated from Riyadh and Jeddah (P1-P4 and P11). Five toxicity patterns were found in the Riyadh isolates and only one toxicity pattern was found in the Jeddah isolates. Six isolates were not harboring any of the toxin genes. Isolates from Riyadh were found to have the highest toxin gene patterns, and for this reason they were used for quantitative real-time PCR.

### Real-Time PCR for Determination of Relative Gene Expression of Toxin Genes

**Fig. 3:**
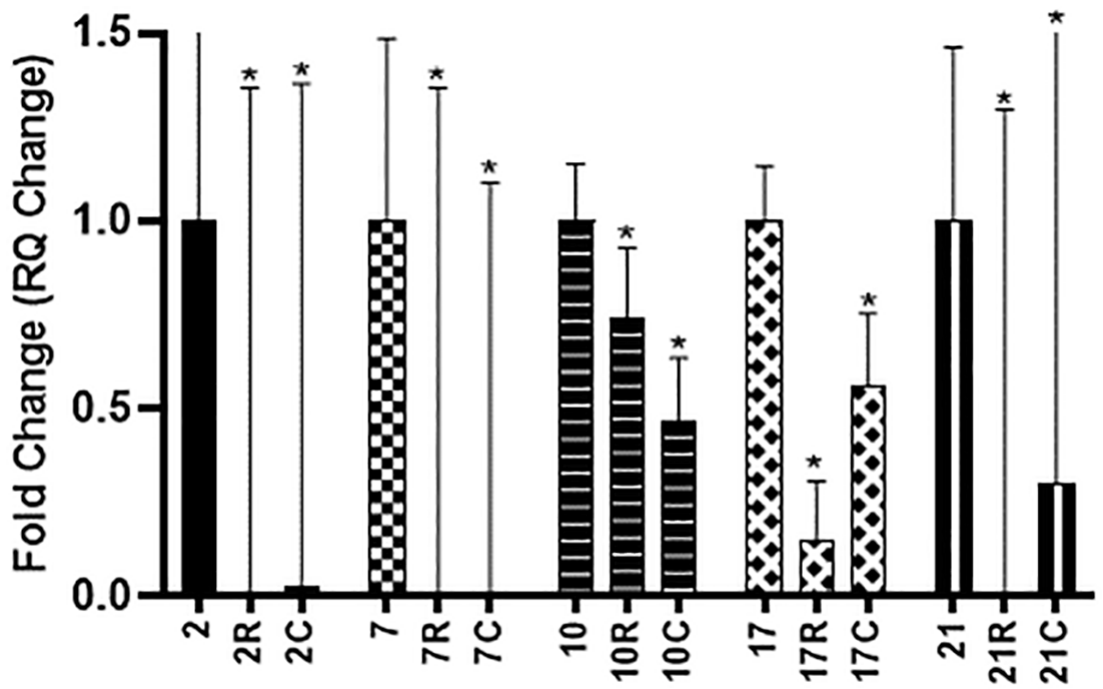
Effect of RSV (R) and CUR (C) on relative gene expression of *sea* gene in *sea* positive MRSA isolates (2, 7, 10, 17, 21). The effect of each concentration is compared to untreated control.

The relative gene expression using real-time PCR of *lukF*, *lukS*, *sea*, and *seb* genes was performed due to their higher frequency among the tested isolates. Fig. 3 demonstrated the relative expression levels of the *sea* gene in 5 positive *sea* MRSA strains (2, 7, 10, 17, and 21) treated with RSV (50 μg/ml) and CUR (20 μg/ml). It was revealed that among the tested isolates, RSV dramatically decreased the transcription of the *sea* gene by 85–99.9%. In addition, CUR downregulated the level of transcription of the *sea* gene in all tested isolates by 43–99.9%.

**Fig. 4:**
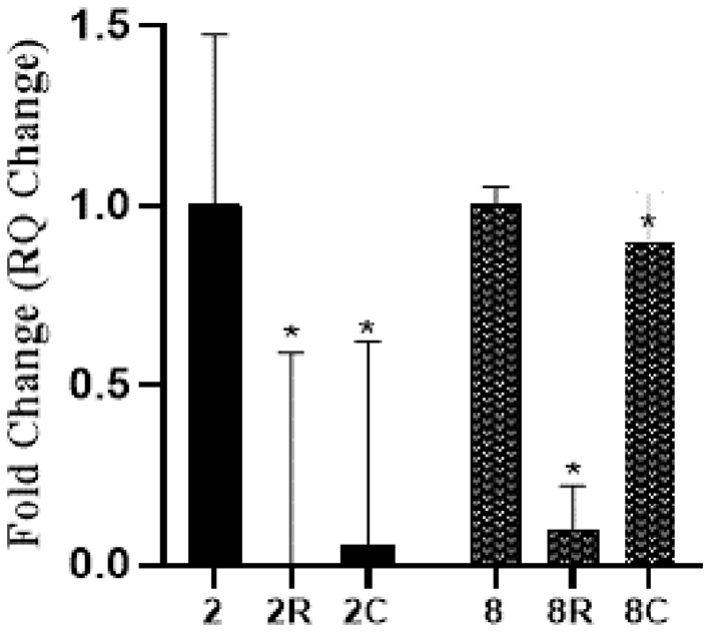
Effect of RSV (R) and CUR (C) on relative gene expression of *seb* gene in *seb* positive MRSA isolates (2, 8). The effect of each concentration is compared to untreated control.

Both RSV (50 μg/ml) and CUR (20 μg/ml) decreased the expression of *seb* gene with different degrees on the tested MRSA strains. RSV reduced the *seb* expression by 90–99.6%. CUR downregulated the transcription with 94.5% in strain No. 2, while the reduction was only 10% in strain No. 8 as shown in Fig. 4.

**Fig. 5:**
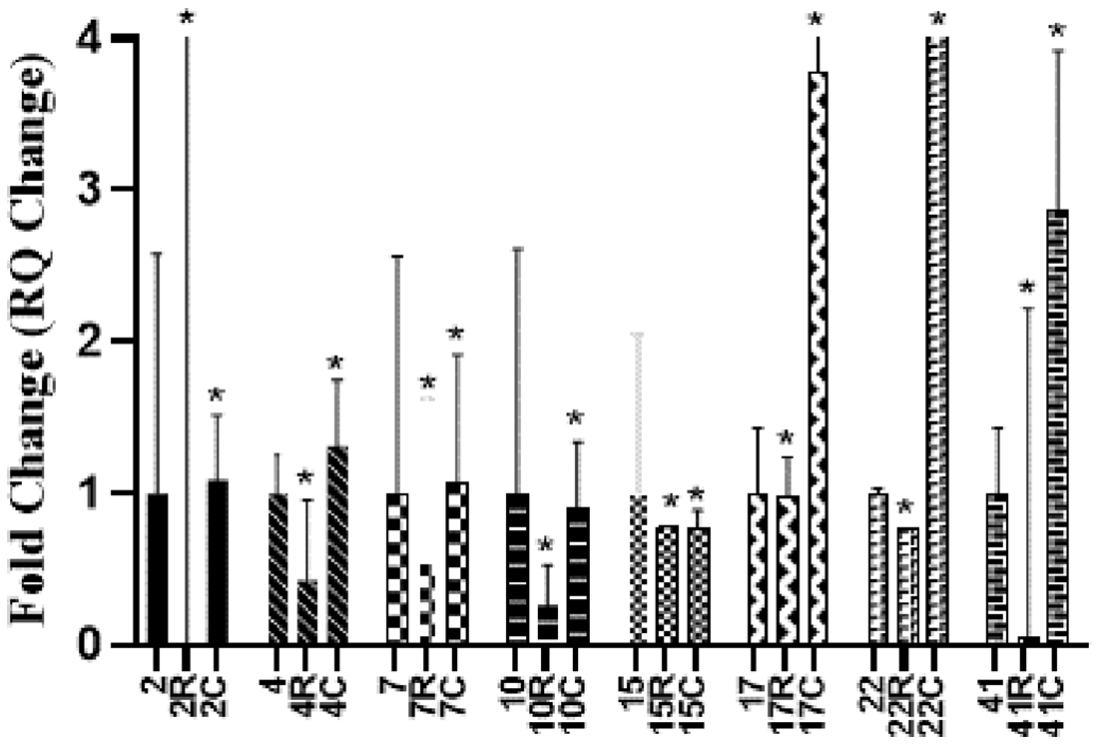
Effect of RSV (R) and CUR (C) on relative gene expression of *lukF* gene in *lukF* positive MRSA isolates (2, 4, 7, 10, 15, 17, 22, 41). The effect of each concentration is compared to untreated control.

The relative expression levels of *lukF* gene of 8 positive *lukF* MRSA isolates (2, 4, 7, 10, 15, 17, 22, 41) after incubation with RSV and CUR was monitored. Fig. 5 showed that RSV significantly reduced the transcription of *lukF* gene among the tested isolates ranging from 1.7–99.8%, while CUR upregulated the level of transcription of *lukF* gene in all tested isolates except isolates 10 and 15, in which CUR downregulated the transcription by 9.3%and 23% respectively.

**Fig. 6:**
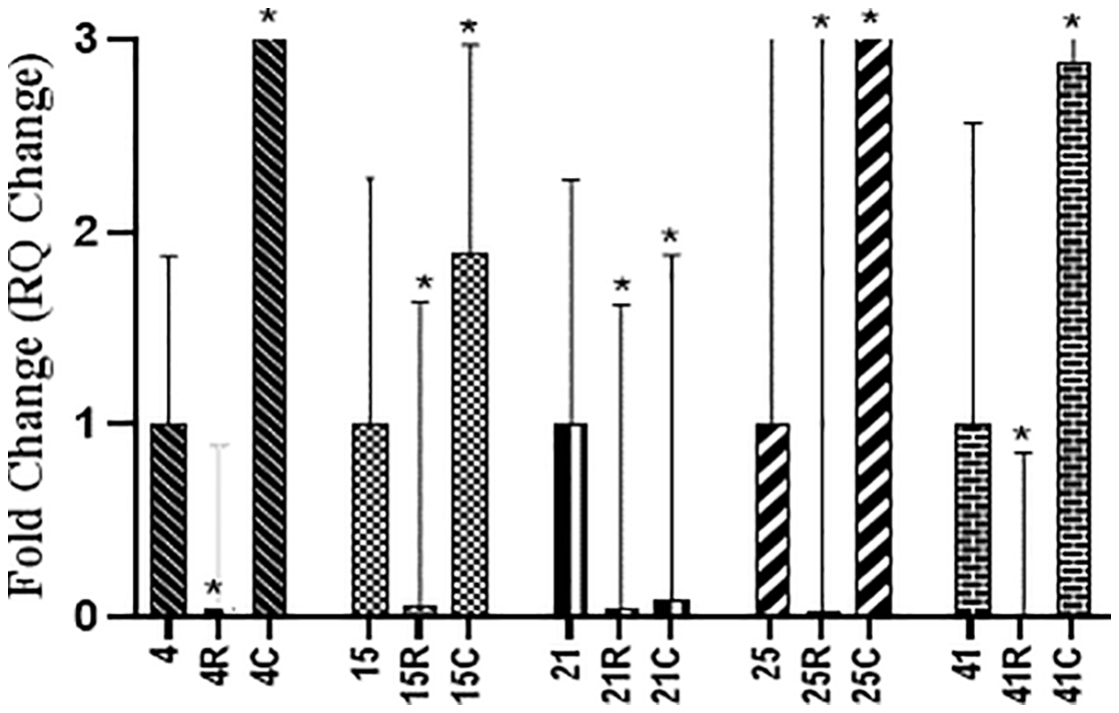
Effect of RSV (R) and CUR (C) on relative gene expression of *lukS* gene in *lukS* positive MRSA isolates (4, 15, 21, 25, 41). The effect of each concentration is compared to untreated control.

Fig. 6 showed that RSV significantly downregulated the gene expression of *lukS* gene in the five tested isolates by 95–100%. CUR decreased gene expression only in isolate 21, while upregulating the expression in the other tested isolates.

## Discussion

*Staphylococcus aureus* is one of the main bacterial infections that affect humans and cause a wide range of clinical symptoms. Due to the rise of multi-drug resistance strains like MRSA, infections are widespread in both community- and hospital-acquired settings, and treatment is still challenging to manage. Most healthy people have *S. aureus* on their skin and mucous membranes, usually in the nose region. *S. aureus* is also present in the environment and in typical human flora. Normal healthy skin does not become infected by *S. aureus*; but, if the germs are permitted to penetrate the bloodstream or internal tissues, they may cause a number of potentially harmful illnesses. Direct contact is usually how transmission occurs, while other means of transmission cause various other illnesses. The prevalence of virulent, drug-resistant MRSA strains has exacerbated the morbidity and mortality brought on by *S. aureus* infection [21]. On the one hand, it is believed that *S. aureus* may be able to create a chronic infection through the secretion of a large quantity of toxins [22].

Because of their therapeutic properties, plant-derived compounds have been used to treat a wide range of human diseases. Polyphenolic chemicals obtained from several plant sources, including flavonoids and phenolic acids, show antimicrobial activity against a diversity of microorganisms, and provide useful antibacterial tools for natural warfare [23].

RSV a polyphenolic substance found in numerous plant extracts, possesses a range of biological activities, including anti-inflammatory, anti-cancer, and antibacterial actions [24]. Several studies suggest that RSV inhibits the expression of toxins [25]. RSV inhibits the endocytosis of the cholera toxin (CT) into host cells for *Vibrio cholerae* and specifically binds CT, potentially preventing the diarrhea that CT causes [26]. Consequently, RSV greatly lowers *S. aureus* in human blood cells [27].

Curcumin, which gives turmeric its yellow color, is also a polyphenolic compound. Multiple pharmacological properties of CUR have been reported including antimicrobial, antidiabetic, anti-inflammatory, anticancer, and antioxidant properties [28]. The first studies on CUR biological action were based on its antibacterial activity against *S. aureus*, *Trichophyton gypseum*, *Salmonella paratyphi*, and *Mycobacterium tuberculosis* [29]. Curcumin's ability to combat MSSA and MRSA has been established in recent years [30]. Curcumin decreases the development of bacterial biofilms, host receptor attachment, and other virulence factors by bacteria thanks to their quorum sensing regulatory mechanism [31].

This is the first investigation into how RSV and CUR affect the expression of several *S. aureus* toxins in Saudi Arabia. Our results showed that RSV and CUR have the ability to downregulate the toxin production at sub-MICs of RSV (50 μg/ml) and CUR (20 μg/ml). Such attributes may be important in reducing the virulence of MRSA strains and could be used to combat the resistance of MRSA to antibiotics, either if those natural compounds are used alone or in combination with antibiotics.

Numerous human cases of food poisoning and infections of the soft tissues and bones, as well as potentially fatal toxic shock, are caused by *S. aureus*. Staphylococcal enterotoxins (SEs) are among the virulence factors that are produced by this ubiquitous bacterium. Along with the SEs that activate particular T cell subsets [32], other virulence factors secreted by *S. aureus* include adhesins, collagenases, protein A, coagulases, hemolysins, and leukocidins [33]. MRSA's pathogenicity is increased by the leukotoxin Panton-Valentine leukocidin (PVL), which can also result in severe necrotic pneumonia. PVL is coded by the toxins *lukS*-PV and *lukF*-PV [34].

Many phenolic substances and extracts can block the synthesis and/or action of bacterial enterotoxins, even at concentrations below the MIC [35]. These phenolic substances can influence the production of enterotoxins in a variety of ways, including by inhibiting translation and/or transcription, disabling secretory pathways, impairing quorum sensing regulatory systems, and toxin inactivation [36].

Resveratrol and curcumin were effective against the tested MRSA isolates in this study with MICs of 500 and 125 μg /ml, respectively. The results regarding RSV’s MIC are in line with those of Duan *et al*. [22], who claimed that the compound's MIC value against four strains of different ST-type *S. aureus* strains was 512 μg/ml. According to Su *et al*. [37], the RSV’s MIC range for each of the 34 used strains obtained from blood was 500–1,000 μg/ml. A study using 10 isolates of *S. aureus* obtained from Wonkwang University Hospital and from the Culture Collection of Antimicrobial Resistant Microbes (CCARM) revealed that the MIC of CUR ranged from 125 to 250 μg/ml [38]. In agreement with our results, Kali *et al*. [39] found that the CUR MIC against 15 gram-positive bacteria, including two *Enterococcus faecalis* and thirteen *S. aureus* strains, was 126.9 μg/ml.

Screening of MRSA toxin genes was done using uniplex PCR for detection of *sea*, *Seb*, *sec*, *sed*, *seh*, *lukF*, and *lukS* genes. The results revealed that the most predominant gene among the tested genes was *lukF* gene that was found in 44 (88%) of isolates with equal distribution between the Riyadh and Jeddah isolates. The least gene detected was *sed* gene detected only in 2 (4%) strains as shown in Fig. 1. The profile of toxin genes was done based on analysis of PCR results; we investigated 11 toxin gene patterns as illustrated in Table 1. Five patterns were common for strains isolated from Riyadh and Jeddah (P1-P4 and P11). The Riyadh isolates exhibited five toxicity patterns, while only one toxicity pattern was found in the Jeddah isolates.

Resveratrol and curcumin were used in sub-MIC concentrations (50 and 20 μg/ml, respectively) to determine their effect on the relative expression levels of toxin genes. Regarding enterotoxin genes *sea* and *seb*, the inhibitory effect of RSV on both genes was significant among the tested isolates and ranged from 85–99.9% for *sea* gene and from 90–99.6% for *seb* gene. Curcumin downregulated the level of transcription of the *sea* gene in all tested isolates from 43–99.9%, while for *seb* gene, CUR downregulated the transcription of the two tested stains with 94.5% in strain No. 2 and only 10% is strain No. 8. Our findings are consistent with those of Shimamura *et al*. [40], who claimed that RSV significantly reduced the expression of the *sea* gene in *S. aureus* C-29. According to Kadhim *et al*.[41], RSV treatment reversed all inflammatory markers, and liver damage caused by *seb* was reduced significantly. These findings support earlier research indicating that RSV is a strong anti-inflammatory drug that can shield mice from acute lung injury caused by *seb* [42]. Bisdemethoxycurcumin (BDMC), an analogue of CUR, was studied by Wang *et al*. [43] for its ability to suppress the MRSA enterotoxins A and B. In *S. aureus* ATCC 33591, the transcription levels of both *sea* and *seb* decreased by 1.7-fold and 5-fold, respectively, leading to a dose-dependent substantial downregulation of the expression of both proteins.

The effect of RSV and CUR on leukocidin genes was also studied, and the results showed that CUR significantly increased the expression of both *lukF* and *lukS* genes in the majority of tested isolates, while RSV significantly decreased the expression of both genes in those isolates. Due to its impact on leukocytes and resistance to phagocytosis, the capacity of RSV to lower *lukF*/S gene expression is a crucial property that helps the pathogen survive longer in tissues during infections. Previous studies only described the effect of RSV on the gene expression of the *hly* gene, therefore the effect of those two compounds on the gene expression of PV genes was explored for the first time [22].

We conclude that RSV and CUR, two naturally occurring substances derived from plants, exhibit an inhibitory effect on the gene expression of MRSA toxin at sub-MIC levels. Resveratrol inhibited gene expression of the tested toxin genes by downregulating their transcription, while CUR showed less effect on the gene expression of the tested genes. More work is needed to study the effect of CUR on toxin production. Finally, the results revealed that both compounds could be used as candidate drugs for the treatment of MRSA infections as an innovative, therapeutic approach.

## Figures and Tables

**Table 1:** Primer sequences of the tested genes.

Gene	Sequence	Amplicon size	Reference
*sea*	F 5'-AGCTTGTATGTATGGTGGTGT-3' R 5'-ACGTCTTGCTTGAAGATCCA-3'	172	[18]
*seb*	F 5'-AGGACACTAAGTTAGGGAAT-3' R 5'-CTCAGTTACACCACCATACA-3'	200	[18]
*sec*	F 5'-TGTAGGTAAAGTTACAGGTGGT-3' R 5'-TGTCTAGTTCTTGAGCTGTTAC-3'	182	[18]
*sed*	F 5'-GTTTGATTCTTCTGATGGGTCT-3' R 5-GAAGGTGCTCTGTGGATAATG-3'	119	[18]
*seh*	F 5'-TGCGAAAGCAGAAGATTTACAC-3' R 5'-TCATTGCCACTATCACCTTGA-3	165	[18]
*lukF*	F 5'-TGTGCTTCTACTTTCCACCAT-3' R 5'-TGTGACTGACTTTTGCACCA-3'	225	[19]
*lukS*	F 5'-GGTCCATCAACAGGAGGTAAT-3' R 5'-AGGATTGAAACCACTGTGTACT-3'	267	[19]
*nuc*	F 5'-GCGATTGATGGTGATACGGTI-3' R 5'AGCCAAGCCTTGACGAACTAAAGC-3'	267	[20]

F, forward; R, reverse

**Table 2:** Toxin gene patterns identified among MRSA isolates.

Pattern No.	Toxin profile	Riyadh isolates	Jeddah isolates	Total	*p*-value
P1	*lukF-PV*	4	9	13	0.1854
P2	*lukF-PV*, *lukS-PV*	2	4	6	0.6640
P3	*sea*, *lukF-PV*	2	3	5	1.0000
P4	*seb*, *lukF-PV*	4	2	6	0.6640
P5	*sea*, *sec*, *lukF-PV*	2	0	2	0.4884
P6	*sea*, *sed*, *lukF-PV*	2	0	2	0.4884
P7	*sea*, *seh*, *lukF-PV*	1	0	1	1.0000
P8	*sea*, *lukF-PV*, *lukS-PV*	3	0	3	0.2326
P9	*seb*, *seh*, *lukF-PV*	1	0	1	1.0000
P10	*sec*, *lukF-PV*, *lukS-PV*	0	2	2	0.4884
P11	*seh*, *lukF-PV*, *lukS-PV*	1	2	3	1.0000
		22	22	44	
